# Psychosocial Predictors of Infant and Young Child Feeding Practices Among Mother‐Child Dyads in Malawi and South Africa

**DOI:** 10.1111/mcn.70045

**Published:** 2025-05-14

**Authors:** Taryn J. Smith, Chikondi Mchazime, Pious Makaka, Giulia Ghillia, Donna Herr, Marlie Miles, Chloë Jacobs, Sadeeka Williams, Thandeka Mazubane, Zayaan Goolam Nabi, Michal R. Zieff, Emmie Mbale, Melissa J. Gladstone, Kirsten A. Donald

**Affiliations:** ^1^ Department of Women's and Children's Health, Institute of Life Course and Medical Sciences University of Liverpool Liverpool UK; ^2^ Department of Paediatrics and Child Health Kamuzu University of Health Sciences Blantyre Malawi; ^3^ Department of Paediatrics and Child Health University of Cape Town Cape Town South Africa; ^4^ Neuroscience Institute University of Cape Town Cape Town South Africa

**Keywords:** breastfeeding, complementary feeding, dietary diversity, intimate partner violence, low‐ and middle‐income countries, maternal mental health, social support, stimulation practices

## Abstract

Maternal capacity to adhere to recommended infant and young child feeding (IYCF) practices may be influenced by psychosocial factors. However, research examining associations between psychosocial factors and IYCF practices, and in particular complementary feeding indicators, is limited. As part of the Khula birth cohort study, we aimed to investigate associations between maternal depression, exposure to intimate partner violence (IPV), social support and stimulating home environments with IYCF practices among mother‐child dyads in Malawi (*n* = 153) and South Africa (*n* = 255). When children were 10–16 months of age, mothers completed a series of psychosocial and child diet questionnaires. Regression modelling assessed associations between maternal psychosocial measures and IYCF indicators, adjusting for maternal age, education, marital status and household socioeconomic status. IYCF practices were suboptimal in both settings, with 50%–54% meeting the minimum dietary diversity (MDD), 67%–73% the minimum meal frequency (MMF) and 39%–45% the minimum acceptable diet (MAD) indicators. In South Africa, mothers exposed to IPV in the previous 12 months were less likely to meet the MDD and MAD recommendations (MDD: OR 0.38, 95% CI: 0.19, 0.75; *p* = 0.006; MAD: OR 0.41, 95% CI: 0.20, 0.85; *p* = 0.02). There was a significant positive association between stimulation (i.e., more books/toys/play activities) and dietary diversity scores in South Africa. In adjusted analyses, maternal depression and social support were not significantly associated with IYCF indicators in either setting. IYCF programmes may benefit from supporting maternal psychosocial wellbeing and integrating nurturing care to improve children's dietary intakes, growth and development.

## Summary

- Maternal psychosocial factors may affect a mother's ability to optimally feed her child; these factors are potentially modifiable through intervention.
- Feeding practices were suboptimal in both Malawi and South Africa.
- Mothers exposed to intimate partner violence in the previous 12 months were less likely to feed their child a minimally diverse or minimally acceptable diet.
- More stimulation reported within the home environment was positively associated with children's dietary diversity.
- Interventions aimed at enhancing maternal psychosocial wellbeing and integrating nurturing care into existing infant and young child feeding programmes may benefit children's dietary intakes, growth and development.

## Graphical abstract

We examined the associations between maternal psychosocial measures and infant and young child feeding practices in Malawi and South Africa. Mothers in South Africa recently exposed to intimate partner violence were less likely to feed their child a minimally diverse or minimally acceptable diet. We found a significant positive association between stimulation and dietary diversity. IYCF programmes may benefit from supporting maternal psychosocial wellbeing and integrating nurturing care to improve children's dietary intakes.

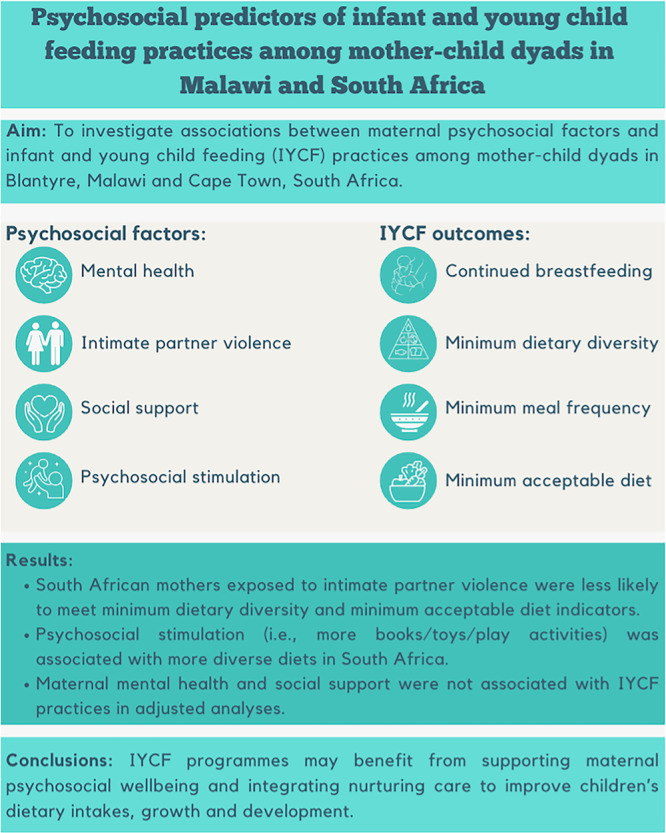

## 1 Introduction

Ensuring adequate nutrition is critical for children's growth and development. Suboptimal feeding practices in the first 2 years of life, including inadequate breastfeeding, quantity and diversity of foods, are risk factors for poor child health, growth and development (Black et al. 2013). From 6 months of age onwards, breastmilk alone is insufficient to meet the high nutritional requirements to support infant and young children's growth and development, and the World Health Organization (WHO) recommends the introduction of nutrient‐dense and safe complementary foods, together with continued breastfeeding up to 2 years of age (World Health Organization and United Nations Children's Fund 2021). The WHO has several indicators for measuring infant and young child feeding (IYCF) practices, including minimum dietary diversity (MDD), minimum meal frequency (MMF) and minimum acceptable diet (MAD) (World Health Organization and United Nations Children's Fund 2021).

Despite the well‐recognised benefits of optimal IYCF practices, a significant proportion of infants and young children 6–23 months of age in low‐ and middle‐income countries (LMICs) are not fed according to WHO recommendations (Gatica‐Domínguez et al. 2021). Examinations of IYCF practices in 32 countries in sub‐Saharan Africa where Demographic and Health Survey data were available between 2010 and 2020 demonstrated IYCF practices that would put a child at risk of poor nutrition among large proportions of respondents. Continued breastfeeding at 1 year (12–15 months of age) was reasonably high (87.2%), but decreased to 50.7% at 2 years (20–23 months) (Zong et al. 2021). Among children 6–23 months of age, the prevalence of MDD, MMF and MAD was 25.3%, 41.2% and 13.3%, respectively (Aboagye et al. 2023). Specifically in Malawi, while a high proportion of children are breastfed at 1 year (87.9%), low proportions of children meet the MDD (25.1%), MMF (29.2) and MAD (8.1%) indicators (National Statistical Office Malawi and ICF 2017). Conversely, in South Africa, complementary feeding indicators are met by more children (MDD: 49.3%; MMF: 51.9%; MAD: 22.9%), but only half are breastfed at 1 year (National Department of Health, Statistics South Africa, & South African Medical Research Council and ICF 2019).

The sociodemographic predictors of IYCF practices are well described. Greater maternal education and age, utilisation of health services and increased household wealth are associated with optimal feeding practices (Aboagye et al. 2023; Kang et al. 2023; Mekonen et al. 2024; Shaker‐Berbari et al. 2021). Psychosocial factors are potentially modifiable factors that may also be implicated in a mother's ability to meet recommended IYCF practices. The mechanisms linking poor psychosocial wellbeing (e.g., experience of poor mental health or intimate partner violence (IPV)) and IYCF practices are likely to be multifactorial but may include impaired decision‐making, cognitive focus and motivation to adhere to optimal feeding practices, lower self‐esteem or mothering self‐efficacy, isolation from social networks and lack of social support and reduced autonomy limiting access to services for IYCF counselling (Slomian et al. 2019; Woldetensay et al. 2021). An estimated 18.6% of women in sub‐Saharan Africa experience postpartum depression (Woldeyohannes et al. 2021), and 38% and 27% of ever‐married/partnered women (15–49 years) in Malawi and South Africa, respectively, have experienced IPV in their lifetime (National Department of Health, Statistics South Africa, & South African Medical Research Council and ICF 2019; National Statistical Office Malawi and ICF 2017). Research examining associations between psychosocial factors and IYCF practices, particularly complementary feeding indicators, is limited and previous research has largely focused on breastfeeding indicators in the first 6 months postpartum. For example, pre‐ and postnatal depression and lifetime or prenatal exposure to IPV have been associated with decreased timely initiation of breastfeeding, lower odds of exclusive breastfeeding and early cessation of breastfeeding (Adewuya et al. 2008; Kaiyo‐Utete et al. 2023; Misch and Yount 2014; Walters et al. 2021). On the other hand, positive psychosocial factors, such as social support, can help to mitigate some of the impacts of psychosocial risk and promote optimal IYCF practices (Frith et al. 2017).

Responsive caregiving and early learning opportunities, as highlighted by the Nurturing Care Framework, are essential for children to develop to their full potential (World Health Organization, United Nations Children's Fund, & World Bank Group 2018). Responsive feeding, as part of responsive caregiving, provides an opportunity for infants and young children to interact with their caregivers in a stimulating family eating environment and for positive caregiver verbalisations towards the child (Black and Aboud 2011). Evidence suggests that responsive feeding practices may help improve nutrient and complementary food intake, and positive caregiver verbalisations during feeding can increase child acceptance of foods (Bentley et al. 2011). Therefore, more stimulating home environments may potentially improve IYCF practices. However, evidence from LMICs, particularly in sub‐Saharan Africa, suggests a low proportion of caregivers engage in high levels of stimulation with their children (Cuartas et al. 2020).

The widespread occurrence of psychosocial risk across contexts in sub‐Saharan Africa, coupled with the potential for modifying these factors through intervention, presents an opportunity for maternal psychosocial support and nurturing care to be integrated into existing IYCF programmes. However, the impact of psychosocial factors on continued breastfeeding and complementary feeding in settings in Malawi and South Africa is currently unknown. Thus, the aim of this study was to investigate associations between maternal depression, exposure to IPV, social support and stimulation within the home with IYCF practices among mothers and their young children in Malawi and South Africa. Understanding how modifiable maternal psychosocial factors influence IYCF practices may help identify barriers that mothers encounter that may reduce the effectiveness of interventions. This knowledge can guide the design of interventions that more effectively support maternal psychosocial wellbeing and nurturing care to improve child nutrition.

## 2 Methods

### 2.1 Study Design, Setting and Population

This is a secondary analysis of data collected from mother‐child dyads who participated in a longitudinal birth cohort study, the Khula Study, in Blantyre, Malawi and Cape Town, South Africa. The primary aim of the study was to characterise brain development, specifically the emergence of executive functions, over the first 1,000 days of life across diverse cultures and geographies. The study protocol has previously been described in detail (Zieff et al. 2024). Briefly, women who were pregnant (28–36 weeks' gestation) or up to 3 months postpartum and > 18 years of age were eligible to participate. Women with multiple pregnancy, psychotropic drug use during pregnancy, infant congenital malformations and abnormalities (e.g., spina bifida, Down's syndrome) and significant delivery complications (e.g., birth asphyxia) were excluded. Women were recruited from antenatal clinics in and around Blantyre district in southern Malawi and Gugulethu, a township in the Western Cape Province of South Africa, between December 2021 and August 2023. Mother‐child dyads were invited for study visits at five time points between 3 and 24 months of age for a range of assessments, including sociodemographic, child health, developmental, behavioural, neuroimaging, maternal psychosocial, child sleep and biospecimen sampling. This secondary analysis uses psychosocial indicators and child feeding data collected at the third study visit when children were approximately 10–16 months of age.

### 2.2 Maternal Psychosocial Indicators

#### 2.2.1 Maternal Mental Wellbeing

The Edinburgh Postnatal Depression Scale (EPDS) was used to assess maternal mental wellbeing. The EPDS is a brief 10‐item tool to screen women for postnatal depression (Cox et al. 1987). Mothers respond to statements about the frequency of depressive symptoms over the previous 7 days using a 4‐point Likert scale (0–3). Scores are obtained by totalling individual scores, with higher scores indicating a higher number and frequency of depressive symptoms (possible range 0–30). A score of ≥ 9 indicates a high risk of depression (Tsai et al. 2013). The EPDS has been validated in both Malawi (Stewart et al. 2013) and South Africa (Lawrie et al. 1998).

#### 2.2.2 Intimate Partner Violence (IPV)

Exposure to IPV was constructed from separate questions about recent (past 12 months) exposure to forms of physical, sexual and emotional abuse and controlling behaviours by an intimate partner (Garcia‐Moreno et al. 2006). Exposure to IPV was defined as mothers reporting at least one form of physical, sexual or emotional abuse in the previous 12 months.

#### 2.2.3 Social Support

Social support was assessed by the Multidimensional Scale of Perceived Social Support (MSPSS). The MSPSS is a brief 12‐item questionnaire to measure the respondent's perception of the adequacy of support they receive from three different sources: a significant other, family, and friends (Zimet et al. 1988). The original version of the MSPSS includes 7 possible responses to each statement ranging from ‘strongly disagree’ to ‘strongly agree’ (scored 0–6). However, the MSPSS was adapted to the local contexts by reducing the number of response options on the Likert scale from 7 to 5 (scored 0–4; possible total score 0–48) (Stewart et al. 2014). A higher score indicates greater perceived social support.

#### 2.2.4 Psychosocial Stimulation

The Family Care Indicators (FCI) tool was used to assess stimulation and caregiver interaction within the home. The FCI is a brief tool developed by UNICEF that measures opportunities for stimulation and learning within the home environment of children < 5 years of age (Kariger et al. 2012). Items are clustered into four subscales: (i) availability of children's books within the household (yes/no); (ii) sources of play materials (score 0–3); (iii) variety of play materials (score 0–7); (iv) play activities with caregivers in the previous 3 days (score 0–6). Scores were calculated for each subscale and a total FCI score as the sum for all four subscales (range 0–17), with a greater score indicating more stimulation and caregiver interaction with the child.

### 2.3 Infant and Young Child Feeding (IYCF) Indicators

IYCF practices were based on mothers' recall of foods and beverages her child consumed in the previous 24 h using structured survey questions (World Health Organization and United Nations Children's Fund 2021). Continued breastfeeding was determined as the number of children who were receiving breastmilk at around 1 year of age. MDD was calculated as the proportion of children who received food from ≥ 5 out of 8 defined food groups the previous day. The eight food groups were: (i) breastmilk; (ii) grains, roots and tubers; (iii) pulses, nuts and seeds; (iv) milk and dairy products; (v) meat, poultry and fish; (vi) eggs; (vii) vitamin A rich fruits and vegetables; (viii) other fruits and vegetables. Dietary diversity score was the number of food groups consumed the previous day (range 0–8). MMF was the proportion of children who received solid, semi‐solid or soft foods at least the minimum number of times the previous day (three times for breastfed and four times for non‐breastfed children). MAD was calculated as the proportion of children who consumed the MDD and the MMF in the previous 24 h.

### 2.4 Socioeconomic and Child Health Indicators

Self‐reported indicators of socioeconomic status (SES) collected at enrolment included education and occupation of the mother, household size and composition, housing characteristics, access to utilities and household ownership of assets. A household SES score was calculated as the number of indicators available in the household from the following: (i) electricity; (ii) improved water source; (iii) improved sanitation; (iv) man‐made flooring (Malawi) or type of dwelling (South Africa); (v) improved cooking fuel; (vi) ownership of household assets. A score of 1 was assigned if the indicator was available in the household and 0 if not. Scores were summed for a total SES score (range 0–6). Child recumbent length and weight were assessed at the third study visit following standard protocols (Cashin and Oot 2018). Age‐ and sex‐specific z‐scores were calculated using WHO growth standards (World Health Organization 2006).

### 2.5 Statistical Analysis

Data were collected and managed using REDCap electronic data capture tools hosted at the University of Cape Town and subsequently analysed in SPSS version 29.0.1.0 (IBM SPSS Statistics, Armonk, NY). Descriptive statistics are presented as mean ± standard deviation or median (Q1, Q3) for continuous variables and frequencies (percentages) for categorical variables. Unadjusted and adjusted regression analyses were conducted to identify which maternal psychosocial measures (EPDS, IPV, MSPSS and FCI [total and subscale scores]) were significantly associated with IYCF indicators (continued breastfeeding, MDD, MMF, MAD and dietary diversity score). Regression models included all four maternal psychosocial measures. Adjusted analyses controlled for maternal age, maternal education (none/primary/started secondary vs. completed secondary or above) and marital status (married/cohabiting vs. single/divorced/widowed) as binary variables and continuous household SES score. Separate analyses were run for each country. Results are presented as odds ratio (OR) and 95% confidence interval (CI) or β and 95% CI. All tests were two‐sided at a 5% level of significance.

### 2.6 Ethical Approval

Ethical approval was obtained from the College of Medicine Research Ethics Committee at the Kamuzu University of Health Sciences, Malawi (Ref. P.11/21/3463) and the Human Research Ethics Committee at the University of Cape Town, South Africa (Ref. 666/2021). Informed consent was obtained from one primary caregiver, usually the mother, after a detailed explanation of the study in a language appropriate to the family.

## 3 Results

### 3.1 Characteristics of Mothers and Their Children

A total of 153 and 255 mother‐child dyads in Malawi and South Africa, respectively, with complete data for all outcome and exposure variables were included in the analysis. Maternal and household characteristics at enrolment are presented in Table 1. Mothers had a mean ± SD age of 27.0 ± 6.0 years in Malawi and 29.4 ± 5.7 years in South Africa. The majority (83.7%) of mothers in Malawi were married, while in South Africa over half (65.1%) were single/never married. Almost all (97.3%) mothers in South Africa had some secondary education or above compared to 60.8% in Malawi. Mothers in Malawi were largely employed (46.4%) or housewives (40.5%), while over half (54.9%) of mothers in South Africa were unemployed and just over a third (34.9%) were employed.

**Table 1:** Maternal and household characteristics at enrolment.^a^

Characteristics	Malawi (*n* = 153)	South Africa (*n* = 255)
Age (years)	27.0 ± 6.0	29.4 ± 5.7
Marital status, *n* (%)		
Married	128 (83.7)	43 (16.9)
Cohabiting	—	45 (17.6)
Single	13 (8.5)	166 (65.1)
Divorced/separated	10 (6.5)	1 (0.4)
Widowed	2 (1.3)	0 (0.0)
Gravidity, *n* (%)		
1	28 (18.3)	51 (20.0)
2	44 (28.8)	85 (33.3)
≥ 3	61 (39.9)	119 (46.7)
Missing	20 (13.1)	0 (0.0)
Education, *n* (%)		
No formal education	8 (5.2)	0 (0.0)
Started primary	30 (19.6)	0 (0.0)
Completed primary	22 (14.4)	7 (2.7)
Started secondary	44 (28.8)	118 (46.3)
Completed secondary	38 (24.8)	98 (38.4)
Some/completed postsecondary education	11 (7.2)	32 (12.5)
Occupation, *n* (%)		
Unemployed	11 (7.2)	140 (54.9)
Housewife	62 (40.5)	10 (3.9)
Farmer	5 (3.3)	—
Employed	71 (46.4)	89 (34.9)
Student	4 (2.6)	16 (6.3)
Household SES score^b^	5 (4, 5)	4 (3, 6)
Improved drinking water source, *n* (%)^c^	140 (91.5)	191 (74.9)
Improved sanitation, *n* (%)^d^	115 (75.2)	107 (42.0)
Household size	4 (4, 5)	4 (3, 6)
Number of children (0–17 years) in the household	2 (1, 3)	2 (1, 3)

Abbreviation: SES, socioeconomic status.

^a^ Values represent mean ± SD or median (Q1, Q3) for continuous variables or n (%) for categorical variables.

^b^ Household SES is the number of indicators available in the household from the following: electricity, improved water source, sanitation, man‐made flooring (Malawi) or type of dwelling (South Africa), improved cooking fuels, and ownership of household assets (range 0–6).

^c^ Improved drinking water sources were defined as those protected from outside contamination.

^d^ Improved sanitation facilities were defined as those that hygienically separate human waste from human contact.

Maternal psychosocial measures are presented in Table 2. Mothers in South Africa had a higher median (Q1, Q3) EPDS score than those in Malawi (4 (0, 9) and 2 (0, 6), respectively). Additionally, 28.2% of South African mothers had an EPDS ≥ 9, indicative of a high risk of depression, compared to 4.6% of mothers in Malawi. More mothers in South Africa had been exposed to any form of IPV in the previous 12 months than in Malawi (20.4% and 11.8%, respectively). Levels of perceived social support were similar between the two countries, with slightly higher levels of support reported from a significant other than from friends or family. FCI total score and household books, sources and varieties of play materials subscale scores were higher in South Africa. However, mothers in Malawi reported more caregiver interaction and play activities with adults in the previous 3 days, particularly for telling stories, singing songs, taking the child outside and playing with the child (Supporting Information: Table 1).

**Table 2:** Child characteristics, infant and young child feeding (IYCF) indicators and maternal psychosocial measures.^a^

Characteristics	Malawi (*n* = 153)	South Africa (*n* = 255)
**Child characteristics**		
Age (mon)	13.3 ± 1.7	14.2 (13.2, 14.6)
Male, *n* (%)	77 (50.3)	130 (51.0)
Length‐for‐age z‐score	−1.1 (−2.0, −0.6)	−0.7 ± 1.2
Weight‐for‐age z‐score	−0.4 (−1.0, 0.4)	0.5 ± 1.3
Weight‐for‐length z‐score	0.5 (−0.5, 1.2)	1.1 ± 1.3
**IYCF indicators**		
Continued breastfeeding, *n* (%)	149 (97.4)	113 (44.3)
Dietary diversity score (0–8)	5 (4, 6)	5 (4, 6)
Minimum dietary diversity, *n* (%)	83 (54.2)	129 (50.6)
Minimum meal frequency, *n* (%)	112 (73.2)	170 (66.7)
Minimum acceptable diet, *n* (%)	69 (45.1)	99 (38.8)
**Maternal psychosocial measures**		
EPDS score	2 (0, 6)	4 (0, 9)
EPDS ≥ 9	7 (4.6)	72 (28.2)
IPV exposure in the past 12 months, *n* (%)		
Yes	18 (11.8)	52 (20.4)
No	135 (88.2)	203 (79.6)
MSPSS total score (0–48)	37 (34, 42)	34 (28, 40)
Significant other score (0–16)	13 (12, 16)	14 (12, 16)
Family score (0–16)	12 (12, 15)	12 (9, 15)
Friends score (0–16)	12 (11, 13)	10 (4, 12)
FCI total score (0–17)	6 (5, 8)	9 (7, 12)
FCI subscale scores		
Household children's books (yes), *n* (%)	39 (25.5)	83 (32.5)
0	114 (74.5)	172 (67.5)
1–2	32 (20.9)	49 (19.2)
≥ 3	7 (4.6)	34 (13.3)
Sources of play materials (0–3)^b^	1 (0, 2)	2 (2, 3)
0, *n* (%)	41 (26.8)	20 (7.8)
1, *n* (%)	50 (32.7)	32 (12.5)
2, *n* (%)	62 (40.5)	100 (39.2)
3, *n* (%)	0 (0.0)	103 (40.4)
Varieties of play materials (0–7)^c^	1 (0, 1)	3 (2, 5)
0, *n* (%)	60 (39.2)	20 (7.8)
1–2, *n* (%)	79 (51.6)	65 (25.5)
3–4, *n* (%)	14 (9.2)	104 (40.8)
≥ 5, *n* (%)	0 (0.0)	66 (25.9)
Play activities (0–6)^d^	4 (4, 4)	3 (3, 4)
0, *n* (%)	0 (0.0)	11 (4.3)
1–2, *n* (%)	5 (3.3)	35 (13.7)
3–4, *n* (%)	111 (72.5)	147 (57.6)
5–6, *n* (%)	37 (24.2)	62 (24.3)

Abbreviations: EPDS, Edinburgh Postnatal Depression Scale; FCI, Family Care Indicators; IPV, Intimate Partner Violence; IYCF, infant and young child feeding; MSPSS, Multidimensional Scale of Perceived Social Support.

^a^ Values represent mean ± SD or median (Q1, Q3) for continuous variables or n (%) for categorical variables.

^b^ Homemade toys, shop bought toys, household objects.

^c^ Things/toys that play or make music, things for drawing or writing, picture books for children, things meant for stacking/constructing/building, things for moving around, toys for learning shapes and colours, things for pretending.

^d^ Read books or looked at picture books with child, told stories to child, sang songs to child, took child outside the home, played with child, counted or drew things with child.

Among children (Malawi: 13.3 ± 1.7 months; South Africa: 14.2 (13.2, 14.6) months), 97.4% were still being breastfed in Malawi, compared to 44.3% in South Africa (Table 2). The median dietary diversity score was the same in both Malawi and South Africa (5 (4, 6)), with similar proportions of children meeting the MDD recommendation (Malawi: 54.2%; South Africa: 50.6%). The proportion of children consuming each food group in the previous 24 h is shown in Supporting Information: Figure 1. In both settings, intake of grains, roots and tubers was > 96%, while egg consumption was ≤ 16%. Milk and dairy products (driven by formula milk intake), meat, poultry and fish and other fruit and vegetable intake was higher in South Africa, while more Malawian children consumed pulses, nuts and seeds and vitamin A rich fruit and vegetables. The proportion of children consuming animal source foods (eggs, meat, fish, poultry) the previous day was 62.1% in Malawi and 78.4% in South Africa. In Malawi, 28.1% of children consumed zero fruit or vegetables the previous day, compared to 16.9% in South Africa. The prevalence of MMF was greater in Malawi than South Africa (73.2% and 66.7%, respectively), although in both countries less than half of the children were fed a minimally acceptable diet (Malawi: 45.1%; South Africa: 38.8%).

### 3.2 Associations Between Maternal Psychosocial Measures and IYCF Practices

Associations between maternal psychosocial measures and continued breastfeeding, MDD, MMF and MAD are presented in Table 3. In Malawi, mothers with higher EPDS scores were less likely to continue breastfeeding (OR 0.72, 95% CI: 0.51, 1.01; *p* = 0.05) in unadjusted analyses. However, this association was attenuated after adjusting for maternal age, education, marital status and household SES (OR 0.70, 95% CI: 0.47, 1.04; *p* = 0.08). There were no other associations between EPDS scores and complementary feeding indicators in Malawi or between maternal depression and any IYCF indicators in South Africa.

**Table 3:** Associations between maternal psychosocial measures and infant and young child feeding practices in unadjusted and adjusted models.^a^

	Continued breastfeeding		MDD		MMF		MAD	
Variables	Unadjusted	Adjusted^b^	Unadjusted	Adjusted^b^	Unadjusted	Adjusted^b^	Unadjusted	Adjusted^b^
**Malawi**								
EPDS	0.72 (0.51, 1.01)*	0.70 (0.47, 1.04)	0.99 (0.89, 1.11)	0.99 (0.89, 1.11)	0.91 (0.81, 1.03)	0.91 (0.80, 1.03)	0.91 (0.83, 1.04)	0.93 (0.83, 1.04)
IPV exposure (yes)	0.68 (0.06, 8.20)	0.43 (0.03, 6.86)	0.51 (0.18, 1.43)	0.57 (0.20, 1.63)	0.85 (0.27, 2.69)	1.04 (0.32, 3.34)	0.78 (0.28, 2.20)	0.91 (0.32, 2.64)
MSPSS total score	1.04 (0.88, 1.23)	1.08 (0.88, 1.33)	1.04 (0.98, 1.10)	1.03 (0.97, 1.09)	0.95 (0.89, 1.02)	0.95 (0.86, 1.01)	1.01 (0.95, 1.07)	1.00 (0.94, 1.06)
FCI total score	0.73 (0.41, 1.32)	0.85 (0.45, 1.61)	1.13 (0.94, 1.36)	1.07 (0.88, 1.30)	1.23 (0.98, 1.55)	1.15 (0.90, 1.05)	1.12 (0.94, 1.34)	1.05 (0.86, 1.27)
**South Africa**								
EPDS	1.04 (0.99, 1.09)	1.03 (0.99, 1.08)	1.04 (0.99, 1.09)	1.05 (0.99, 1.10)	1.02 (0.97, 1.07)	1.02 (0.97, 1.07)	1.04 (0.99, 1.09)	1.05 (0.99, 1.10)
IPV exposure (yes)	0.60 (0.31, 1.16)	0.53 (0.27, 1.06)	0.41 (0.21, 0.81)**	0.38 (0.19, 0.75)**	1.04 (0.52, 2.07)	1.06 (0.53, 2.13)	0.42 (0.21, 0.86)*	
MSPSS total score	1.01 (0.98, 1.04)	1.01 (0.98, 1.04)	1.02 (0.99, 1.05)	1.02 (0.99, 1.05)	1.02 (0.98, 1.05)	1.01 (0.98, 1.05)	1.02 (0.99, 1.06)	1.02 (0.99, 1.06)
FCI total score	0.97 (0.90, 1.05)	0.97 (0.90, 1.05)	1.05 (0.98, 1.14)	1.05 (0.97, 1.13)	1.04 (0.96, 1.13)	1.03 (0.94, 1.12)	1.06 (0.98, 1.14)	1.05 (0.97, 1.13)

Abbreviations: EPDS, Edinburgh Postnatal Depression Scale; FCI, Family Care Indicators; IPV, Intimate Partner Violence; MAD, minimum acceptable diet; MDD, minimum dietary diversity; MMF, minimum meal frequency; MSPSS, Multidimensional Scale Of Perceived Social Support.

a Values represent odds ratio (95% confidence interval).

b Adjusted for maternal age, maternal education (completed secondary education or above, yes vs. no), marital status (married/cohabiting, yes vs. no), and household socioeconomic status score.

* *p* ≤ 0.05;

** *p* ≤ 0.01.

In South Africa, mothers who reported exposure to any form of IPV in the previous 12 months were less likely to meet the MDD and MAD recommendations (MDD: OR 0.41, 95% CI: 0.21, 0.81; *p* = 0.009; MAD: OR 0.42, 95% CI: 0.21, 0.86; *p* = 0.02). These associations remained significant in adjusted analyses (MDD: OR 0.38, 95% CI: 0.19, 0.75; *p* = 0.006; MAD: OR 0.41, 95% CI: 0.20, 0.85; *p* = 0.02). Mothers exposed to IPV were also less likely to continue breastfeeding (OR 0.53, 95% CI: 0.27, 1.06; *p* = 0.07 adjusted analyses). There were no other associations between IPV exposure and any IYCF indicators in Malawi.

MSPSS scores, measuring perceived social support, were not significantly associated with any IYCF indicators in either Malawi or South Africa.

Stimulation within the home environment, indicated by a higher FCI total score, was positively associated with dietary diversity score in unadjusted multiple regression analyses in both Malawi (β 0.17, 95% CI: 0.001, 0.26; *p* = 0.05) and South Africa (β 0.16, 95% CI: 0.01, 0.11; *p* = 0.01) (Table 4). However, this only remained significant in South Africa after adjusting for covariates (β 0.14, 95% CI: 0.01, 0.10; *p* = 0.02).

**Table 4:** Associations between maternal psychosocial measures and dietary diversity score in unadjusted and adjusted analyses.^a^

	Dietary diversity score	
Variables	Unadjusted	Adjusted^b^
**Malawi**		
EPDS	0.01 (−0.08, 0.08)	0.01 (−0.08, 0.08)
IPV exposure	−0.06 (−1.02, 0.47)	−0.05 (−1.01, 0.52)
MSPSS total score	0.05 (−0.03, 0.05)	0.05 (−0.03, 0.06)
FCI total score	0.17 (0.001, 0.26)*	0.15 (−0.02, 0.26)
**South Africa**		
EPDS	0.03 (−0.02, 0.04)	0.05 (−0.02, 0.04)
IPV exposure	−0.11 (−0.76, 0.06)	−0.12 (−0.79, 0.03)
MSPSS total score	0.10 (−0.004, 0.03)	0.09 (−0.01, 0.03)
FCI total score	0.16 (0.01, 0.11)**	0.14 (0.01, 0.10)*

Abbreviations: EPDS, Edinburgh Postnatal Depression Scale; FCI, Family Care Indicators; IPV, Intimate Partner Violence; MSPSS, Multidimensional Scale of Perceived Social Support.

a Values represent β (95% confidence interval).

b Adjusted for maternal age, maternal education (completed secondary education or above, yes vs. no), marital status (married/cohabiting, yes vs. no), and household socioeconomic status score.

* *p* ≤ 0.05

** *p *≤ 0.01.

Malawian mothers who reported more sources of play materials within the home were 1.9 times more likely to meet the MMF recommendation (OR 1.92, 95% CI: 1.17, 3.13; *p* = 0.009; Supporting Information: Table 2). This remained significant in adjusted analyses (OR 1.79, 95% CI: 1.09, 2.93; *p* = 0.02). Conversely, in South Africa, mothers who reported more varieties of play materials within the home were 1.2 times more likely to meet the MDD (OR 1.19, 95% CI: 1.01, 1.40; *p* = 0.04) and MMF (OR 1.20, 95% CI: 1.00, 1.43; *p* = 0.50) recommendations. These associations were attenuated in adjusted analyses (MDD: OR 1.17, 95% CI: 0.99, 1.40; *p* = 0.07; MMF: OR 1.18, 95% CI: 0.98, 1.41; *p* = 0.08). A significant positive association was also found for varieties of play materials and dietary diversity score in both unadjusted (β 0.25, 95% CI: 0.08, 0.28; *p* < 0.001) and adjusted analyses (β 0.23, 95% CI: 0.06, 0.27; *p* = 0.002) in South Africa (Supporting Information: Table 3).

## 4 Discussion

In this study, we examined associations between four potentially modifiable maternal psychosocial factors and IYCF practices among mother‐child dyads in Malawi and South Africa. Findings showed that recent exposure to IPV was associated with reduced odds of meeting MDD and MAD in South Africa. FCI total and subscale scores, as indicators of stimulation within the home environment, were positively associated with dietary diversity, MDD and MMF. Malawian mothers with higher depressive symptoms were less likely to continue breastfeeding at around 1 year, although this did not remain statistically significant after adjusting for sociodemographic factors. Perceived social support was not significantly associated with any IYCF practices within these cohorts.

Consistent with other Malawian and South African studies, we found low rates of MDD and MAD, and low rates of continued breastfeeding in South Africa (Budree et al. 2017; du Plessis et al. 2016; Kuchenbecker et al. 2017). Interestingly, there was a lower prevalence of optimal feeding practices in South Africa than Malawi across all the IYCF indicators. This is in contrast to a recent study of MDD in nine East and South African countries, where South Africa had the greatest proportion of children 6–23 months of age meeting MDD, albeit 38.5%, attributed to greater urbanisation in South Africa (Kang et al. 2023). The difference in the present study may be due to the lower maternal employment, household SES and improved drinking water source in our sample of South African mother‐child dyads than in Malawi, factors associated with lower odds of MDD (Kang et al. 2023).

One of the most salient findings from this study was that South African mothers exposed to IPV in the past 12 months (i.e., the child's lifetime) were less likely to meet the MDD and MAD indicators and less likely to continue breastfeeding at 1 year. A recent rapid evidence assessment of IPV and child nutrition outcomes identified only two studies that examined feeding practices beyond breastfeeding (Bhatt Carreno et al. 2024). In agreement with findings of the present study, both studies, conducted in Ethiopia, found that maternal experience of IPV was associated with lower odds of MAD (Tsedal et al. 2020) and poorer infant feeding practices (Woldetensay et al. 2021). While IPV has been associated with lower odds of early initiation of breastfeeding and exclusive breastfeeding, and earlier cessation of exclusive breastfeeding (Ariyo and Jiang 2021; Kjerulff Madsen et al. 2019; Misch and Yount 2014; Walters et al. 2021), we are aware of only one other study which has considered continued breastfeeding at 1 year (Walters et al. 2021). In that study, Tanzanian mothers who often or sometimes experienced emotional IPV were two times more likely not to continue breastfeeding to 1 year compared to mothers reporting no experience of emotional IPV. However, similar to the lack of association in Malawi in the present study, Walters et al. did not find similar associations in Malawi or Zambia. Thus, the relationship between maternal experience of IPV and continued breastfeeding remains unclear. Lifetime exposure to IPV among women increases the odds of wasting and stunting among their children < 5 years (Bhatt Carreno et al. 2024; Dadi et al. 2024). However, the pathways through which maternal exposure to IPV impacts child nutritional status have not been clearly elucidated (Bhatt Carreno et al. 2024). Taking together the findings from these studies, it is plausible that suboptimal feeding practices are one pathway linking IPV exposure and child undernutrition. Further research examining these associations in different contexts is needed. Screening for IPV during IYCF counselling, as well as during antenatal care as already recommended by the WHO (World Health Organization 2016), may help mitigate the effects of IPV on IYCF practices (Frith et al. 2017).

Another important finding was the positive associations between FCI total score and the sources and varieties of play materials subscale scores and MDD, MMF and dietary diversity score. We are aware of only one other study which examined associations between the FCI and IYCF indictors, and similarly found FCI total and subscale scores were positively associated with MDD, MMF and MAD among children 24 months of age in the Democratic Republic of the Congo, Guatemala and Pakistan, but not in India (Long et al. 2024). These findings suggest that households that reported more play, stimulation and caregiver interaction with their young children fed their children more diverse diets. This highlights a valuable opportunity for integrating nurturing care into IYCF counselling and these positive associations should be further examined in larger, diverse populations. In general, IYCF programmes inform caregivers on what to feed infants and young children, but rarely how to feed them. Incorporating nurturing care into existing IYCF programmes, such as responsive caregiving, responsive feeding and early learning opportunities through play and stimulation, can improve caregiver behaviours, with the potential to improve child growth and development, as has been recently demonstrated in Ghana and the Kyrgyz Republic (Aidam et al. 2024; Oot et al. 2024).

In the current study, we found little evidence that maternal mental health was associated with IYCF practices. Mothers in Malawi with higher EPDS scores were less likely to continue breastfeeding at around 1 year, although this did not remain statistically significant after adjusting for sociodemographic factors. Previous studies in Nigeria, Brazil and Pakistan have reported greater cessation of breastfeeding within the first 12 months postpartum among mothers with depressive symptoms (Adewuya et al. 2008; Feldens et al. 2012; Taj and Sikander 2003). However, it is important to consider that in Malawi we found a high adherence to continued breastfeeding at 1 year (97.4%) and prevalence of depressive symptoms was low. No significant association was found in South Africa where continued breastfeeding was low (44.3%) and mothers reported greater depressive symptoms. Pregnancy and postpartum depression have been associated with shorter breastfeeding duration and early complementary feeding in systematic reviews (Dias and Figueiredo 2015; Slomian et al. 2019). However, most of the included studies were conducted in high‐income countries and measured breastfeeding outcomes before 12 months postpartum. Maternal mental health problems occurring during pregnancy and postpartum constitute a serious public health problem and threat to child health and development, especially in LMICs (Gelaye et al. 2016), and globally, the prevalence of continued breastfeeding at 1 and 2 years remains below WHO targets for 2030 (World Health Organization 2023). It is therefore important to understand how maternal mental health problems impact breastfeeding indicators beyond the first 6 months postpartum, and how policies and programmes can support women to continue breastfeeding for longer. In contrast to the present study, two studies in Ethiopia found maternal postpartum depressive symptoms were associated with inappropriate complementary feeding practices (Anato et al. 2020; Woldetensay et al. 2021). The variation in results between contexts suggests that unmeasured contextual factors may influence the association between maternal depression and IYCF practices, and further research should examine these associations in different contexts.

Perceived social support was not associated with any IYCF indicators in either country. Social support has been positively associated with breastfeeding and complementary feeding in Ethiopia, Kenya and Uganda (Basnet et al. 2020; Ickes et al. 2018; Mukuria et al. 2016), but not in Ghana (Wemakor et al. 2022). Across studies, many constructs and scales have been used to measure social support, making comparisons difficult. Furthermore, social support has multiple dimensions: emotional (e.g., trust, caring, understanding), informational (e.g., advice) and instrumental (e.g., aid, services, physical support) (Heaney and Israel 2008). Most studies have measured general social support that may lack specificity and contribute to the inconsistent findings reported between social support and IYCF practices. For example, among Ugandan mothers, greater emotional and informational exclusive breastfeeding‐specific social support was associated with exclusive breastfeeding at 1 and 3 months (Miller et al. 2022). Therefore, future studies should measure IYCF‐specific social support, including domain‐specific social support, to understand how social support influences IYCF practices and pathways through which interventions could impact IYCF practices.

This study has several strengths including the use of continued breastfeeding at 1 year, which has rarely been considered as a breastfeeding indicator, and the use of reliable questionnaires validated in each setting. Furthermore, the maternal psychosocial factors were assessed during a time period relevant to the outcomes of interest. For example, we considered exposure to IPV in the previous 12 months, directly preceding the measurement of the IYCF outcomes. Previous studies examining IPV experience and breastfeeding have rarely documented IPV occurring postnatally in the child's lifetime, but rather a mother's lifetime experience of IPV (Bhatt Carreno et al. 2024). However, this study is not without limitations that must be considered in the interpretation of the findings. Firstly, this analysis was cross‐sectional and thus the findings are not causal. It is also possible that suboptimal IYCF practices may influence maternal psychosocial wellbeing (e.g., stress, anxiety, depression). Future longitudinal studies may provide evidence of temporal associations between maternal psychosocial factors and IYCF practices and child growth and development. Secondly, although we adjusted for maternal‐ and household‐level variables, IYCF practices may also be influenced by maternal knowledge or beliefs, paternal education and occupation, access to health services and cultural norms. Thirdly, this was a secondary analysis of mother‐child dyads enrolled into a longitudinal birth cohort in and around urban Blantyre district in southern Malawi and the township of Gugulethu in the Western Cape, and the results are therefore not generalisable to rural‐dwelling populations in either Malawi or South Africa or populations in other resource‐limited contexts. Finally, all psychosocial factors and children's dietary intakes relied on maternal self‐reported measures and are therefore subject to recall and social desirability bias.

## 5 Conclusion

In conclusion, this study demonstrates that children from diverse communities in Malawi and South Africa had suboptimal feeding practices. We found evidence that recent experience of IPV and possibly maternal depressive symptoms limit a mother's ability to optimally feed her child. For interventions and programmes to be effective in improving IYCF practices in resource‐limited settings, the potential negative impact of maternal psychosocial factors on breastfeeding and complementary feeding must be considered. Conversely, positive caregiving behaviours providing a stimulating home environment with opportunities for early learning are associated with more optimal feeding practices. This presents a valuable opportunity to enhance maternal psychosocial wellbeing and integrate the Nurturing Care Framework into existing IYCF programmes, including responsive care, opportunities for early learning, safety and security (e.g., IPV prevention, screening and referral), and maternal mental wellbeing, alongside child health and nutrition. Future research should examine the longitudinal associations between maternal psychosocial factors and IYCF practices in diverse contexts and evaluate interventions that integrate nurturing care into IYCF programmes and the short‐ and long‐term impacts on child diet quality, growth and development.

## Author Contributions

K.A.D., M.J.G. and E.M. conceived and designed the study protocol; P.M., C.J. and S.W. implemented the data collection; T.J.S., C.M., G.G. and M.R.Z. planned and supervised data collection; D.H. and M.M. were responsible for project management; T.M., Z.G.N. and M.R.Z. performed data cleaning and curation; T.J.S. conceptualised the analyses presented here, analysed the data and wrote the manuscript; all authors contributed to the interpretation of the results, critically reviewed and approved the final version of the manuscript.

## Conflicts of Interest

The authors declare no conflicts of interest.

## Supporting information

Supporting information.

## Data Availability

The data that support the findings of this study can are available upon reasonable request.

## References

[mcn70045-bib-0001] Aboagye, R. G. , A.‐A. Seidu , B. O. Ahinkorah , et al. 2023. “Prevalence and Predictors of Infant and Young Child Feeding Practices in Sub‐Saharan Africa.” International Health 16, no. 1: 68–82. 10.1093/inthealth/ihad022. PMC10759300 37042267

[mcn70045-bib-0002] Adewuya, A. O. , B. O. Ola , O. O. Aloba , B. M. Mapayi , and J. A. O. Okeniyi . 2008. “Impact of Postnatal Depression on Infants' Growth in Nigeria.” Journal of Affective Disorders 108, no. 1: 191–193. 10.1016/j.jad.2007.09.013. 17997163

[mcn70045-bib-0003] Aidam, E. , V. Varela , F. Abukari , et al. 2024. “Promoting Responsive Care and Early Learning Practices in Northern Ghana: Results From a Counselling Intervention Within Nutrition and Health Services.” Public Health Nutrition 27, no. 1: e77. 10.1017/S1368980024000156. 38328894 PMC10966855

[mcn70045-bib-0004] Anato, A. , K. Baye , Z. Tafese , and B. J. Stoecker . 2020. “Maternal Depression Is Associated With Child Undernutrition: A Cross‐Sectional Study in Ethiopia.” Maternal & Child Nutrition 16, no. 3: e12934. 10.1111/mcn.12934. 31833231 PMC7296785

[mcn70045-bib-0005] Ariyo, T. , and Q. Jiang . 2021. “Intimate Partner Violence and Exclusive Breastfeeding of Infants: Analysis of the 2013 Nigeria Demographic and Health Survey.” International Breastfeeding Journal 16, no. 1: 15. 10.1186/s13006-021-00361-9. 33485361 PMC7825158

[mcn70045-bib-0006] Basnet, S. , E. A. Frongillo , P. H. Nguyen , S. Moore , and M. Arabi . 2020. “Associations of Maternal Resources With Care Behaviours Differ by Resource and Behaviour.” Maternal & Child Nutrition 16, no. 3: e12977. 10.1111/mcn.12977. 32216037 PMC7296814

[mcn70045-bib-0007] Bentley, M. E. , H. M. Wasser , and H. M. Creed‐Kanashiro . 2011. “Responsive Feeding and Child Undernutrition in Low‐ and Middle‐Income Countries.” Journal of Nutrition 141, no. 3: 502–507. 10.3945/jn.110.130005. 21270354 PMC3040907

[mcn70045-bib-0008] Bhatt Carreno, S. , M. Orjuela‐Grimm , L. Vahedi , et al. 2024. “Linkages Between Maternal Experience of Intimate Partner Violence and Child Nutrition Outcomes: A Rapid Evidence Assessment.” PLoS One 19, no. 3: e0298364. 10.1371/journal.pone.0298364. 38498450 PMC10947923

[mcn70045-bib-0009] Black, M. M. , and F. E. Aboud . 2011. “Responsive Feeding Is Embedded in a Theoretical Framework of Responsive Parenting.” Journal of Nutrition 141, no. 3: 490–494. 10.3945/jn.110.129973. 21270366 PMC3040905

[mcn70045-bib-0010] Black, R. E. , C. G. Victora , S. P. Walker , et al. 2013. “Maternal and Child Undernutrition and Overweight in Low‐Income and Middle‐Income Countries.” Lancet 382, no. 9890: 427–451. 10.1016/S0140-6736(13)60937-X. 23746772

[mcn70045-bib-0011] Budree, S. , E. Goddard , K. Brittain , S. Cader , L. Myer , and H. J. Zar . 2017. “Infant Feeding Practices in a South African Birth Cohort—A Longitudinal Study.” Maternal & Child Nutrition 13, no. 3: e12371. 10.1111/mcn.12371. 27696743 PMC6866213

[mcn70045-bib-0012] Cashin, K. , and L. Oot . 2018. Guide to Anthropometry: A Practical Tool for Program Planners, Managers and Implementers, 360. Food and Nutrition Technical Assistance III Project (FANTA)/FHI.

[mcn70045-bib-0013] Cox, J. L. , J. M. Holden , and R. Sagovsky . 1987. “Detection of Postnatal Depression: Development of the 10‐Item Edinburgh Postnatal Depression Scale.” British Journal of Psychiatry 150, no. 6: 782–786. 10.1192/bjp.150.6.782. 3651732

[mcn70045-bib-0014] Cuartas, J. , J. Jeong , C. Rey‐Guerra , D. C. McCoy , and H. Yoshikawa . 2020. “Maternal, Paternal, and Other Caregivers' Stimulation in Low‐ and‐ Middle‐Income Countries.” PLoS One 15, no. 7: e0236107. 10.1371/journal.pone.0236107. 32649702 PMC7351158

[mcn70045-bib-0015] Dadi, A. F. , K. Y. Ahmed , Y. Berhane , et al. 2024. “Intimate Partner Violence and Childhood Health Outcomes in 37 Sub‐Saharan African Countries: An Analysis of Demographic Health Survey Data From 2011 to 2022.” Lancet Global Health 12, no. 11: e1785–e1793. 10.1016/S2214-109X(24)00313-9. 39332421

[mcn70045-bib-0016] Dias, C. C. , and B. Figueiredo . 2015. “Breastfeeding and Depression: A Systematic Review of the Literature.” Journal of Affective Disorders 171: 142–154. 10.1016/j.jad.2014.09.022. 25305429

[mcn70045-bib-0017] Feldens, C. A. , M. R. Vitolo , F. Rauber , L. N. Cruz , and J. B. Hilgert . 2012. “Risk Factors for Discontinuing Breastfeeding in Southern Brazil: A Survival Analysis.” Maternal and Child Health Journal 16, no. 6: 1257–1265. 10.1007/s10995-011-0885-7. 21948218

[mcn70045-bib-0018] Frith, A. L. , S. Ziaei , R. T. Naved , A. I. Khan , I. Kabir , and E.‐C. Ekström . 2017. “Breast‐Feeding Counselling Mitigates the Negative Association of Domestic Violence on Exclusive Breast‐Feeding Duration in Rural Bangladesh. The Minimat Randomized Trial.” Public Health Nutrition 20, no. 15: 2810–2818. 10.1017/S1368980017001136. 28659213 PMC10261296

[mcn70045-bib-0019] Garcia‐Moreno, C. , H. A. Jansen , M. Ellsberg , L. Heise , and C. H. Watts . 2006. “Prevalence of Intimate Partner Violence: Findings From the Who Multi‐Country Study on Women's Health and Domestic Violence.” Lancet 368, no. 9543: 1260–1269. 10.1016/S0140-6736(06)69523-8. 17027732

[mcn70045-bib-0020] Gatica‐Domínguez, G. , P. A. R. Neves , A. J. D. Barros , and C. G. Victora . 2021. “Complementary Feeding Practices in 80 Low‐ and Middle‐Income Countries: Prevalence of and Socioeconomic Inequalities in Dietary Diversity, Meal Frequency, and Dietary Adequacy.” Journal of Nutrition 151, no. 7: 1956–1964. 10.1093/jn/nxab088. 33847352 PMC8245881

[mcn70045-bib-0021] Gelaye, B. , M. B. Rondon , R. Araya , and M. A. Williams . 2016. “Epidemiology of Maternal Depression, Risk Factors, and Child Outcomes in Low‐Income and Middle‐Income Countries.” Lancet Psychiatry 3, no. 10: 973–982. 10.1016/S2215-0366(16)30284-X. 27650773 PMC5155709

[mcn70045-bib-0022] Heaney, C. A. , and B. A. Israel . 2008. “Social Networks and Social Support.” In Health Behavior and Health Education: Theory, Research, and Practice, 4th ed., 189–210, edited by K. Glanz , B. K. Rimer , and K. Viswanath . Jossey‐Bass.

[mcn70045-bib-0023] Ickes, S. B. , M. Wu , M. P. Mandel , and A. C. Roberts . 2018. “Associations Between Social Support, Psychological Well‐Being, Decision Making, Empowerment, Infant and Young Child Feeding, and Nutritional Status in Ugandan Children Ages 0 to 24 Months.” Maternal & Child Nutrition 14, no. 1: e12483. 10.1111/mcn.12483. 28782300 PMC6866247

[mcn70045-bib-0024] Kaiyo‐Utete, M. , L. Langhaug , A. Chingono , et al. 2023. “Antenatal Depression: Associations With Birth and Neonatal Outcomes Among Women Attending Maternity Care in Harare, Zimbabwe.” PLoS One 18, no. 7: e0270873. 10.1371/journal.pone.0270873. 37418441 PMC10328234

[mcn70045-bib-0025] Kang, Y. , R. A. Heidkamp , K. Mako‐Mushaninga , et al. 2023. “Factors Associated With Diet Diversity Among Infants and Young Children in the Eastern and Southern Africa Region.” Maternal & Child Nutrition 19, no. 3: e13487. 10.1111/mcn.13487. 36924028 PMC10262889

[mcn70045-bib-0026] Kariger, P. , E. A. Frongillo , P. Engle , P. M. R. Britto , S. M. Sywulka , and P. Menon . 2013. “Indicators of Family Care for Development for Use in Multicountry Surveys.” Journal of Health, Population, and Nutrition 30, no. 4: 472–486. 10.3329/jhpn.v30i4.13417. PMC3763619 23304914

[mcn70045-bib-0027] Kjerulff Madsen, F. , C. E. Holm‐Larsen , C. Wu , et al. 2019. “Intimate Partner Violence and Subsequent Premature Termination of Exclusive Breastfeeding: A Cohort Study.” PLoS One 14, no. 6: e0217479. 10.1371/journal.pone.0217479. 31181090 PMC6557484

[mcn70045-bib-0028] Kuchenbecker, J. , A. Reinbott , B. Mtimuni , M. B. Krawinkel , and I. Jordan . 2017. “Nutrition Education Improves Dietary Diversity of Children 6‐23 Months at Community‐Level: Results From a Cluster Randomized Controlled Trial In Malawi.” PLoS One 12, no. 4: e0175216. 10.1371/journal.pone.0175216. 28426678 PMC5398527

[mcn70045-bib-0029] Lawrie, T. A. , G. J. Hofmeyr , M. de Jager , and M. Berk . 1998. “Validation of the Edinburgh Postnatal Depression Scale on a Cohort of South African Women.” South African Medical Journal = Suid‐Afrikaanse tydskrif vir geneeskunde 88, no. 10: 1340–1344. 9807193

[mcn70045-bib-0030] Long, J. M. , G. Gatica‐Domínguez , J. E. Westcott , et al. 2024. “Infant and Young Child Feeding Indicators Are Positively Associated With Length and Family Care Indicators in the Children of the Women First Trial Participants.” Maternal & Child Nutrition 20, no. 1: e13572. 10.1111/mcn.13572. 37817452 PMC10750017

[mcn70045-bib-0031] Mekonen, E. G. , A. F. Zegeye , and B. S. Workneh . 2024. “Complementary Feeding Practices and Associated Factors Among Mothers of Children Aged 6 to 23 Months in Sub‐Saharan African Countries: A Multilevel Analysis of the Recent Demographic and Health Survey.” BMC Public Health 24, no. 1: 115. 10.1186/s12889-023-17629-w. 38191351 PMC10775555

[mcn70045-bib-0032] Miller, J. D. , S. M. Collins , G. O. Boateng , et al. 2022. “Pathways Linking Social Support, Self‐Efficacy, and Exclusive Breastfeeding Among Women in Northern Uganda.” Global Public Health 17, no. 12: 3506–3518. 10.1080/17441692.2022.2110918. 35960598 PMC9898077

[mcn70045-bib-0033] Misch, E. S. , and K. M. Yount . 2014. “Intimate Partner Violence and Breastfeeding in Africa.” Maternal and Child Health Journal 18, no. 3: 688–697. 10.1007/s10995-013-1294-x. 23807715

[mcn70045-bib-0034] Mukuria, A. G. , S. L. Martin , T. Egondi , A. Bingham , and F. M. Thuita . 2016. “Role of Social Support in Improving Infant Feeding Practices in Western Kenya: A Quasi‐Experimental Study.” Global Health: Science and Practice 4, no. 1: 55–72. 10.9745/GHSP-D-15-00197. 27016544 PMC4807749

[mcn70045-bib-0035] National Department of Health, Statistics South Africa, & South African Medical Research Council and ICF . 2019. South Africa Demographic and Health Survey 2016. NDoH, Stats SA, SAMRC and ICF. http://dhsprogram.com/pubs/pdf/FR337/FR337.pdf.

[mcn70045-bib-0036] National Statistical Office [Malawi] and ICF . 2017. *Malawi Demographic and Health Survey 2015‐16*. Zomba. NSO and ICF. http://dhsprogram.com/pubs/pdf/FR319/FR319.pdf.

[mcn70045-bib-0037] Oot, L. , V. Varela , C. Abdimitalipova , et al. 2024. “Promoting Responsive Care and Early Learning Practices Among Caregivers of Children 0–23 Months in the Kyrgyz Republic: Findings From Integrating a Counselling Intervention With Nutrition Services.” Public Health Nutrition 27, no. 1: e202. 10.1017/S1368980024001642. 39376123 PMC11604327

[mcn70045-bib-0038] du Plessis, L. , M. G. Herselman , M. H. McLachlan , and J. H. Nel . 2016. “Selected Facets of Nutrition During the First 1000 Days of Life in Vulnerable South African Communities.” South African Journal of Child Health 10, no. 1: 37–42.

[mcn70045-bib-0039] Shaker‐Berbari, L. , V. Qahoush Tyler , C. Akik , Z. Jamaluddine , and H. Ghattas . 2021. “Predictors of Complementary Feeding Practices Among Children Aged 6–23 Months in Five Countries in the Middle East and North Africa Region.” Maternal & Child Nutrition 17, no. 4: e13223. 10.1111/mcn.13223. 34137179 PMC8476411

[mcn70045-bib-0040] Slomian, J. , G. Honvo , P. Emonts , J.‐Y. Reginster , and O. Bruyère . 2019. “Consequences of Maternal Postpartum Depression: A Systematic Review of Maternal and Infant Outcomes.” Women's Health 15: 1745506519844044. 10.1177/1745506519844044. PMC6492376 31035856

[mcn70045-bib-0041] Stewart, R. C. , E. Umar , B. Tomenson , and F. Creed . 2013. “Validation of Screening Tools for Antenatal Depression in Malawi—A Comparison of the Edinburgh Postnatal Depression Scale and Self Reporting Questionnaire.” Journal of Affective Disorders 150, no. 3: 1041–1047. 10.1016/j.jad.2013.05.036. 23769290

[mcn70045-bib-0042] Stewart, R. C. , E. Umar , B. Tomenson , and F. Creed . 2014. “Validation of the Multi‐Dimensional Scale of Perceived Social Support (Mspss) and the Relationship Between Social Support, Intimate Partner Violence and Antenatal Depression in Malawi.” BMC Psychiatry 14, no. 1: 180. 10.1186/1471-244X-14-180. 24938124 PMC4074419

[mcn70045-bib-0043] Taj, R. , and K. S. Sikander . 2003. “Effects of Maternal Depression on Breast‐Feeding.” JPMA. The Journal of the Pakistan Medical Association 53, no. 1: 8–11. 12666844

[mcn70045-bib-0044] Tsai, A. C. , J. A. Scott , K. J. Hung , et al. 2013. “Reliability and Validity of Instruments for Assessing Perinatal Depression in African Settings: Systematic Review and Meta‐Analysis.” PLoS One 8, no. 12: e82521. 10.1371/journal.pone.0082521. 24340036 PMC3858316

[mcn70045-bib-0045] Tsedal, D. M. , M. Yitayal , Z. Abebe , and A. T. Tsegaye . 2020. “Effect of Intimate Partner Violence of Women on Minimum Acceptable Diet of Children Aged 6–23 Months In Ethiopia: Evidence From 2016 Ethiopian Demographic and Health Survey.” BMC Nutrition 6, no. 1: 28. 10.1186/s40795-020-00354-7. 32742712 PMC7385870

[mcn70045-bib-0046] Walters, C. N. , H. Rakotomanana , J. J. Komakech , and B. J. Stoecker . 2021. “Maternal Experience of Intimate Partner Violence Is Associated With Suboptimal Breastfeeding Practices in Malawi, Tanzania, and Zambia: Insights From a DHS Analysis.” International Breastfeeding Journal 16, no. 1: 20. 10.1186/s13006-021-00365-5. 33602285 PMC7890985

[mcn70045-bib-0047] Wemakor, A. , V. Awuni , and S. Issah . 2022. “Maternal Autonomy but Not Social Support Is a Predictor of Child Feeding Indicators in the Northern Region, Ghana.” BMC Nutrition 8, no. 1: 135. 10.1186/s40795-022-00630-8. 36401277 PMC9673434

[mcn70045-bib-0048] Woldetensay, Y. K. , T. Belachew , S. Ghosh , E. J. Kantelhardt , H. K. Biesalski , and V. Scherbaum . 2021. “The Effect of Maternal Depressive Symptoms on Infant Feeding Practices in Rural Ethiopia: Community Based Birth Cohort Study.” International Breastfeeding Journal 16, no. 1: 27. 10.1186/s13006-021-00375-3. 33743775 PMC7980325

[mcn70045-bib-0049] Woldeyohannes, D. , Y. Tekalegn , B. Sahiledengle , D. Ermias , T. Ejajo , and L. Mwanri . 2021. “Effect of Postpartum Depression on Exclusive Breast‐Feeding Practices in Sub‐Saharan Africa Countries: A Systematic Review and Meta‐Analysis.” BMC Pregnancy and Childbirth 21, no. 1: 113. 10.1186/s12884-020-03535-1. 33557766 PMC7869485

[mcn70045-bib-0050] World Health Organization . 2006. “The WHO Child Growth Standards: Length/Height and Body Mass Index‐for‐Age.” Methods and Development. https://www.who.int/tools/child-growth-standards.

[mcn70045-bib-0051] World Health Organization . 2016. “WHO Recommendations on Antenatal Care for a Positive Pregnancy Experience.” https://www.who.int/publications/i/item/9789241549912. 28079998

[mcn70045-bib-0052] World Health Organization . 2023. “Global Breastfeeding Scorecard 2023.” https://www.who.int/publications/i/item/WHO-HEP-NFS-23.17.

[mcn70045-bib-0053] World Health Organization and United Nations Children's Fund . 2021. “Indicators for Assessing Infant and Young Child Feeding Practices: Definitions and Measurement Methods.” https://iris.who.int/handle/10665/340706.

[mcn70045-bib-0054] World Health Organization, United Nations Children's Fund, & World Bank Group . 2018. “Nurturing Care for Early Childhood Development: A Framework for Helping Children Survive and Thrive to Transform Health and Human Potential.” https://www.who.int/teams/maternal-newborn-child-adolescent-health-and-ageing/child-health/nurturing-care#:~:text=The%20Nurturing%20Care%20Framework%20is,support%20the%20nurturing%20care%20agenda.

[mcn70045-bib-0055] Zieff, M. R. , M. Miles , E. Mbale , et al. 2024. “Characterizing Developing Executive Functions in the First 1000 Days in South Africa and Malawi: The Khula Study.” Wellcome Open Research 9: 157. 10.12688/wellcomeopenres.19638.1.

[mcn70045-bib-0056] Zimet, G. D. , N. W. Dahlem , S. G. Zimet , and G. K. Farley . 1988. “The Multidimensional Scale of Perceived Social Support.” Journal of Personality Assessment 52, no. 1: 30–41. 10.1207/s15327752jpa5201_2.

[mcn70045-bib-0057] Zong, X. N , H. Wu , M. Zhao , C. G. Magnussen , and B. Xi . 2021. “Global Prevalence of WHO Infant Feeding Practices in 57 LMICs in 2010–2018 and Time Trends Since 2000 for 44 LMICs.” eClinicalMedicine 37: 100971. 10.1016/j.eclinm.2021.100971. 34386748 PMC8343261

