# Supplementary material for: Psychosocial Predictors of Infant and Young Child Feeding Practices Among Mother‐Child Dyads in Malawi and South Africa
Source: Matern Child Nutr. 2025 May 14;21(4):e70045. doi: 10.1111/mcn.70045 (PMC12454209; doi:10.1111/mcn.70045)
Supplement: Supplementary file 1 — Supporting information. [file MCN-21-e70045-s001.docx]

**Table S1.** Family Care Indicator (FCI) subscales in Malawi and South Africa^1^

| **FCI subscale** | **Malawi**  **(n = 153)** | **South Africa**  **(n = 255)** |
| --- | --- | --- |
| **Household books** |  |  |
| None | 114 (74.5) | 172 (67.5) |
| 1-2 | 32 (20.9) | 49 (19.2) |
| ≥3 | 7 (4.6) | 34 (13.3) |
| **Sources of play materials** |  |  |
| Homemade toys | 45 (29.4) | 114 (44.7) |
| Shop bought toys | 90 (58.8) | 213 (83.5) |
| Household objects | 39 (25.5) | 214 (83.9) |
| **Varieties of play materials** |  |  |
| Things/toys that play or make music | 19 (12.4) | 176 (69.0) |
| Things for drawing or writing | 1 (0.7) | 107 (42.0) |
| Picture books for children | 6 (3.9) | 80 (31.4) |
| Things meant for stacking, constructing, building | 2 (1.3) | 75 (29.4) |
| Things for moving around (e.g. balls, tricycles) | 26 (17.0) | 197 (77.3) |
| Toys for learning shapes and colours | 3 (2.0) | 76 (29.8) |
| Things for pretending | 88 (57.5) | 130 (51.0) |
| **Family interaction (play activities in the previous 3 days)** |  |  |
| Read books or looked at picture books with child | 33 (21.6) | 59 (23.1) |
| Told stories to child | 131 (85.6) | 79 (31.0) |
| Sang songs to child | 147 (96.1) | 223 (87.5) |
| Took child outside the home | 151 (98.7) | 203 (79.6) |
| Played with child | 152 (99.3) | 230 (90.2) |
| Counted or drew things with child | 25 (16.3) | 109 (42.7) |

^1^ Values represent n (%).

**Table S2.** Associations between Family Care Indicators subscales and infant and young child feeding practices in unadjusted and adjusted models^1^

| **Variables** | **Continued breastfeeding** | | **MDD** | | **MMF** | | **MAD** | |
| --- | --- | --- | --- | --- | --- | --- | --- | --- |
|  | **Unadjusted** | **Adjusted^2^** | **Unadjusted** | **Adjusted^2^** | **Unadjusted** | **Adjusted^2^** | **Unadjusted** | **Adjusted^2^** |
| **Malawi** |  |  |  |  |  |  |  |  |
| Sources of play materials | 0.28 (0.04, 2.02) | 0.28 (0.03, 2.40) | 1.24 (0.81, 1.87) | 1.16 (0.76, 1.79) | **1.92 (1.17, 3.13)**** | **1.79 (1.09, 2.93)*** | 1.34 (0.88, 2.05) | 1.26 (0.82, 1.96) |
| Varieties of play materials | 1.05 (0.40, 2.77) | 1.25 (0.44, 3.54) | 0.91 (0.65, 1.26) | 0.84 (0.59, 1.20) | 1.26 (0.82, 1.95) | 1.18 (0.75, 1.85) | 0.95 (0.68, 1.33) | 0.89 (0.62, 1.26) |
| Play activities | 0.85 (0.30, 2.47) | 1.06 (0.34, 3.29) | 1.13 (0.78, 1.62) | 1.06 (0.73, 1.53) | 1.02 (0.67, 1.55) | 0.97 (0.63, 1.50) | 1.16 (0.81, 1.66) | 1.08 (0.74, 1.56) |
| **South Africa** |  |  |  |  |  |  |  |  |
| Sources of play materials | 0.97 (0.72, 1.29) | 0.99 (0.73, 1.33) | 0.83 (0.62, 1.11) | 0.83 (0.61, 1.12) | 0.82 (0.59, 1.12) | 0.82 (0.59, 1.13) | 0.84 (0.62, 1.14) | 0.85 (0.63, 1.16) |
| Varieties of play materials | 0.92 (0.78, 1.08) | 0.91 (0.77, 1.08) | **1.19 (1.01, 1.40)*** | 1.17 (0.99, 1.40) | **1.20 (1.00, 1.43)*** | 1.18 (0.98, 1.41) | 1.13 (0.98, 1.40) | 1.13 (0.95, 1.34) |
| Play activities | 1.06 (0.88, 1.28) | 1.08 (0.89, 1.31) | 0.97 (0.80, 1.17) | 0.97 (0.80, 1.18) | 0.93 (0.75, 1.15) | 0.92 (0.74, 1.14) | 1.01 (0.83, 1.23) | 1.01 (0.83, 1.23) |

MDD, minimum dietary diversity; MMF, minimum meal frequency; MAD, minimum acceptable diet

^1^ Values represent odds ratio (95% confidence interval).

^2^ Adjusted for maternal age, maternal education (completed secondary education or above, yes *vs.* no), marital status (married/cohabiting, yes *vs.* no), and household SES.

*p≤0.05; **p≤0.01

**Table S3.** Associations between Family Care Indicator subscales and dietary diversity score in unadjusted and adjusted analyses^1^

| **Variables** | **Dietary diversity score** | |
| --- | --- | --- |
|  | **Unadjusted** | **Adjusted^2^** |
| **Malawi** |  |  |
| Sources of play materials | 0.07 (-0.19, 0.43) | 0.05 (-0.22, 0.41) |
| Varieties of play materials | 0.002 (-0.24, 0.25) | -0.01 (-0.27, 0.24) |
| Play activities | 0.13 (-0.06, 0.47) | 0.11 (-0.09, 0.045) |
| **South Africa** |  |  |
| Sources of play materials | -0.06 (-0.27, 0.10) | -0.06 (-0.27, 0.11) |
| Varieties of play materials | **0.25 (0.08, 0.28)***** | **0.23 (0.06, 0.27)**** |
| Play activities | -0.04 (-0.16, 0.09) | -0.04 (-0.16, 0.08) |

^1^ Values represent β (95% confidence intervals).

^2^ Adjusted for maternal age, maternal education (completed secondary education or above, yes *vs.* no), marital status (married/cohabiting, yes *vs.* no), and household SES.

*p≤0.05; **p≤0.01; ***p<0.001


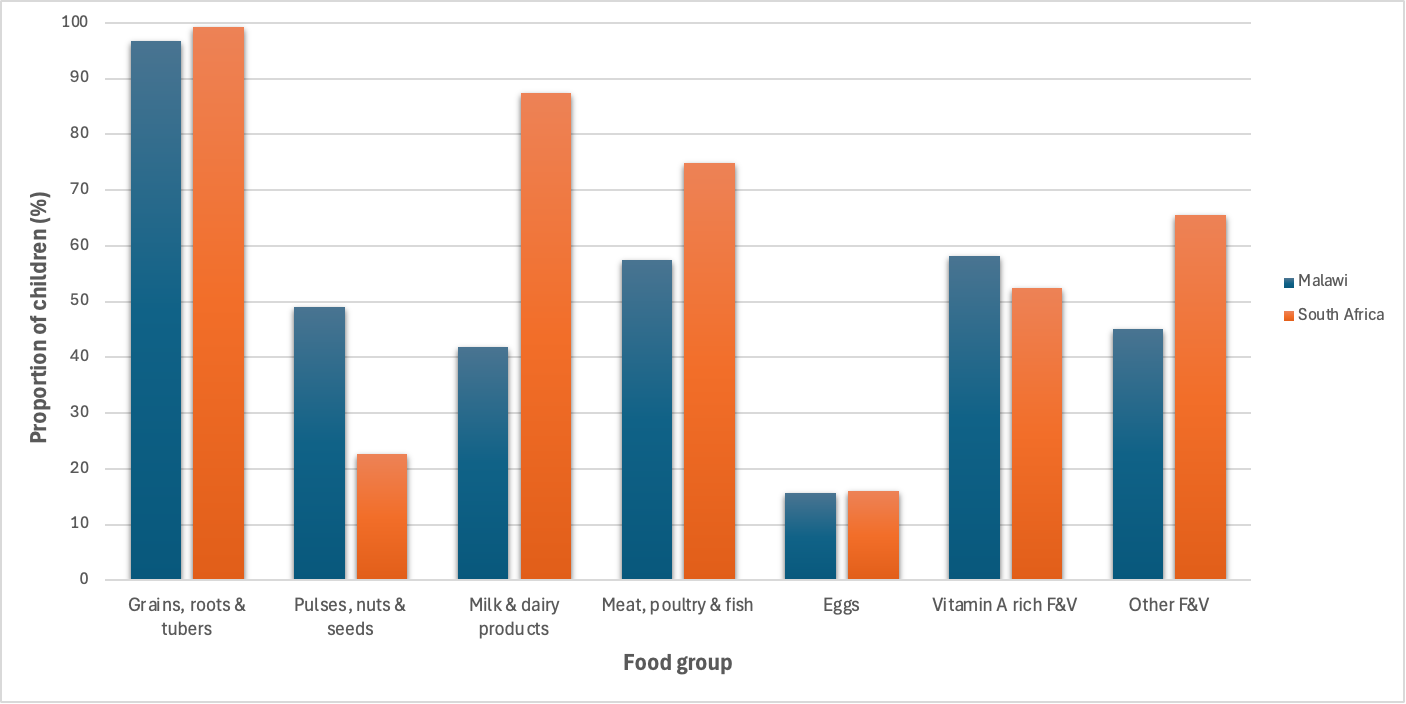


**Figure S1.** Proportion of children in Malawi (n = 153) and South Africa (n = 255) consuming each food group in the previous 24 hours reported by mothers
